# Can group-based reassuring information alter low back pain behavior? A cluster-randomized controlled trial

**DOI:** 10.1371/journal.pone.0172003

**Published:** 2017-03-27

**Authors:** Pernille Frederiksen, Aage Indahl, Lars L. Andersen, Kim Burton, Rasmus Hertzum-Larsen, Tom Bendix

**Affiliations:** 1 Copenhagen Center for Back Research (COPE BACK), Centre for Rheumatology and Spine Diseases, Glostrup, Denmark; 2 Metropolitan University College, Copenhagen, Denmark; 3 Faculty of Health and Medical Sciences, University of Copenhagen, Copenhagen, Denmark; 4 Uni Health, University of Bergen, Bergen, Norway; 5 Department of Research and Development, Clinic Physical Medicine and Rehabilitation, Vestfold Hospital Trust, Stavern, Norway; 6 National Research Centre for the Working Environment, Copenhagen, Denmark; 7 Physical Activity and Human Performance group, Center for Sensory-Motor Interaction, Department of Health Science and Technology, Aalborg University, Aalborg, Denmark; 8 Centre for Applied Psychological and Health Research, University of Huddersfield, Huddersfield, United Kingdom; 9 Danish Cancer Society Research and Data, Copenhagen, Denmark; Vanderbilt University, UNITED STATES

## Abstract

**Background:**

Low back pain (LBP) is common in the population and multifactorial in nature, often involving negative consequences. Reassuring information to improve coping is recommended for reducing the negative consequences of LBP. Adding a simple non-threatening explanation for the pain (temporary muscular dysfunction) has been successful at altering beliefs and behavior when delivered with other intervention elements. This study investigates the isolated effect of this specific information on future occupational behavior outcomes when delivered to the workforce.

**Design:**

A cluster-randomized controlled trial.

**Methods:**

Publically employed workers (n = 505) from 11 Danish municipality centers were randomized at center-level (cluster) to either intervention (two 1-hour group-based talks at the workplace) or control. The talks provided reassuring information together with a simple non-threatening explanation for LBP—the ‘functional-disturbance’-model. Data collections took place monthly over a 1-year period using text message tracking (SMS). Primary outcomes were self-reported days of cutting down usual activities and work participation. Secondary outcomes were self-reported back beliefs, work ability, number of healthcare visits, bothersomeness, restricted activity, use of pain medication, and sadness/depression.

**Results:**

There was no between-group difference in the development of LBP during follow-up. Cumulative logistic regression analyses showed no between-group difference on days of cutting down activities, but increased odds for more days of work participation in the intervention group (OR = 1.83 95% CI: 1.08–3.12). Furthermore, the intervention group was more likely to report: higher work ability, reduced visits to healthcare professionals, lower bothersomeness, lower levels of sadness/depression, and positive back beliefs.

**Conclusion:**

Reassuring information involving a simple non-threatening explanation for LBP significantly increased the odds for days of work participation and higher work ability among workers who went on to experience LBP during the 12-month follow-up. Our results confirm the potential for public-health education for LBP, and add to the discussion of simple versus multidisciplinary interventions.

## Introduction

Low back pain (LBP) is an episodic symptom experienced by most people across the life course. It is a dominant cause of disability [1], long-term sickness absence and early retirement in economically developed countries [2–6]. In respect of back pain experienced at work, relevant guidelines promote a biopsychosocial approach that seeks to accommodate workers with LBP rather than removing them from work [7, 8]. This transition to a biopsychosocial perspective on LBP presents a difficulty for healthcare professionals when they are unable to respond to patients’ expectations of a biomedically-based diagnosis. This leads to an inability to effectively explain the condition, which in turn can lead to patients having unhelpful feelings of uncertainty, worry and even helplessness or hopelessness [9]. This suggests that there will be some benefit from providing a novel biological explanation for why the person is experiencing the pain; a simple explanation that is believable, non-threatening, and consistent with the notion of continuing to participate in activity and work.

A plausible biological component behind non-specific LBP, based on human and porcine studies on the spinal neuromuscular function, has been suggested: LBP can result from a temporary functional disturbance involving abnormal paraspinal muscle activation [10]. Termed a ‘functional disturbance’-model (or in most studies: the ‘non-injury’-model), this explanation was consistent with the emerging concepts present in clinical guidelines that most LBP is not due to permanent spinal damage or disc degeneration [11–14] and that leisure time physical activity and work exposures explain only a small part of degenerative conditions [15, 16]. Thus, the ‘functional disturbance’-model suggests that natural muscle use during leisure and work activities, which is necessary for movement and postural support, will rarely impart harmful spinal loads. Furthermore, the model is based on the studies showing altered muscle activity in persons with LBP (e.g. paraspinal muscles) [10, 17, 18] and that these alterations are somewhat influenced by a persons’ beliefs about the association between movement and occurrence of pain [19, 20]. Therefore, an important element in intervention studies based on the model has been information addressing peoples’ beliefs about pain and movement [21–24].

Arguably, a simple non-threatening explanation for the pain may contribute to reduce the uncertainty of engaging in physical activity and thus creating a positive circle of movement and pain reduction. In that way the model is consistent with, and supplementary to, other positive messages around spinal strength, resumption of activity, and early return to work, which form the basis of modern information and advice intended to promote helpful beliefs about LBP [25–30].

The delivery of information and advice about LBP can be implemented in various ways, at various times, focused on different outcomes. Population-level delivery through a wide-ranging public health campaign has been shown to improve beliefs of both the general population [27, 28, 31] and health professionals [27, 31], and can influence medical management and reduce disability and workers’ compensation costs [27, 32]. Delivery of that same set of messages has been delivered at the workplace through a simple pamphlet broadcast to the entire workforce, which showed a positive effect on beliefs and a modest reduction in extended absence [25]. The same messages have also been delivered just to patients with LBP (as distinct from a mixed population), and showed positive results on clinical outcomes such as pain, fear, beliefs, and disability [26, 29, 30]. The information and advice in all these studies was based around the modern positive approach, but did not include the plausible biomedical explanation for the pain (‘functional disturbance’) proposed by Indahl [10].

Evidence from three intervention-studies using the ‘functional-disturbance’-model have also shown significant improvement in peoples’ back beliefs and reduction in LBP-related disability, including sickness absence [21, 22, 24]. Subsequent follow-up studies have argued for long-term effects as well [33, 34]. So, it would seem that a common set of messages giving positive information and advice about LBP can have beneficial effects on numerous clinical and participation outcomes, including behaviors as well as beliefs, when delivered in a variety of ways both to patients consulting with back pain, and to populations that include people with and without current back pain. The delivery methods have involved public media, pamphlets, booklets, and talks. However, the unique contribution of the ‘functional-disturbance’-message remains unconfirmed. Previous studies have tested the effects of either clinical (three) or workplace (one) interventions combining patient information based on the ‘functional-disturbance’-model with other elements (e.g. clinical examination or workplace peer-support) [21–24].

The aim of this study was to test the isolated effect that information based on a ‘functional-disturbance’ model might have on beliefs and LBP-related behavior, including work participation, in a public workplace setting. The delivery method was in the form of talks to all workers, irrespective of whether they had back pain or not. Thus, the intention of the experimental intervention was to change beliefs and behavior (activity and occupational outcomes), not to influence symptoms or clinical outcomes. The target population for the study was public sector workers, predominantly manual workers, known to be at relatively high risk of sickness absence [35]. The hypothesis was that the intervention would result in improved back beliefs across the target population, along with improved LBP-related behavior in those workers who experienced LBP during the year following the intervention.

## Methods

This was a cluster randomized controlled trial, with a 12-months follow-up period. Technical and environmental centers from five municipalities localized in the Eastern part of Denmark agreed to participate in the study. A-priori criterion for municipality participation was that their technical end environmental department was organized in at least two separate centers (a fixed group of employees of varying size performing a unique set of duties) with minimal daily communication or cooperation but with about the same working tasks to enable an intervention and a control group.

**Fig 1:**
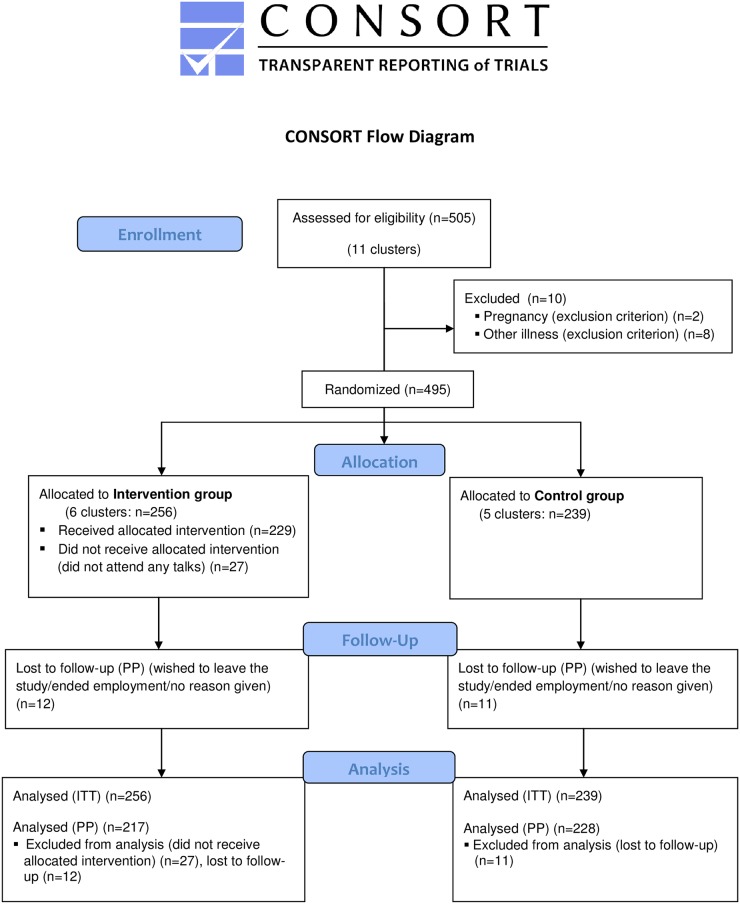
CONSORT flow diagram.

**Fig 2:**
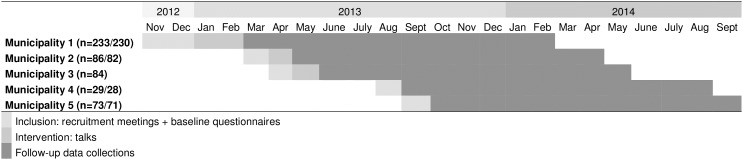
Dates and periods for inclusion and follow-up (n = 505/495) in the five municipalities.

In total, approx. 900 employees, across 11 centers (five municipalities participating with two or in one case three centers each), were invited to recruitment meetings of which 505 consented to participate (Fig 1). Only the research team had access to the names of the attendees who had agreed to participate. The primary investigator (PF) performed all tasks related to recruiting municipalities and participants, and arranging follow-up. Recruitment and baseline assessments took place step-wise between 19^th^ of November 2012 and 23^rd^ of September 2013 (Fig 2) as we realized that more municipalities needed to be included to reach the necessary number of participants. The step-wise inclusion was also due to municipalities having varying wishes for when to enter the study. Manual workers as well as some administrative personnel and line managers were invited to participate. Recruitment involved receiving written information as well as participation in a recruitment meeting at the workplace during the work day.

### Randomization

The randomization of the 11 centers (clusters) took place at each municipality after the last recruitment meeting had taken place. Because municipalities were included stepwise, the randomization also took place step-wise. The randomization involved the primary investigator and one or two municipal representatives. It was highly simple and did not involve computerized procedures. Using two identical closed envelopes each containing a paper with either “intervention” or “control”, a blinded municipal representative placed the envelopes on a table next to signs with the two involved centers’ names on them. In the municipality participating with three centers, one center was randomized using only one sign. The envelope(s) were then opened and the result was verified in writing by the primary investigator and one of the representatives on behalf of the participating municipality.

### Inclusion/Exclusion of participants

The inclusion criteria for participating workers were employment at one of the municipality centers, adequate proficiency to read and write in Danish, and agreement to participate. Exclusion criteria were serious specific LBP (e.g. Morbus Bechterew. Disc herniation and Morbus Scheuermann OK), post-surgery or -trauma LBP, illness seriously affecting the person (e.g. current cancer) at baseline, and illness/pain conditions severely affecting the individual (e.g. acute RA, Lupus), and pregnancy during the first nine months of the study.

### Intervention

The intervention for the experimental group consisted of two one-hour talks held at the workplace, delivered by the primary investigator (PF). The initial talk took place as soon as possible after the last recruitment meeting, with the second taking place approximately two weeks after the first.

The ‘functional-disturbance’-model is based on the following key assumptions [10]:

- The extensive muscular network in the lumbar region and the complex innervation suggests a function with a delicate biomechanical task of load distribution and load transfer
- Non-specific back pain is not a sign of injury–it is likely a sign of a temporary disturbance in the muscle coordination, which may naturally occur in the human body as it ages (e.g. disc degeneration or arthritis) or is temporarily subjected to altered movement patterns for some reason. Locally, the disturbance resembles muscle cramps and should be treated the same way (light activity, normal movement patterns, stretching, cold/heat, massage etc.) until the disturbance has resolved and the person has recovered. The pain can go from acute to long-lasting if the person responds to it by changing their normal movement pattern into highly guarded and very careful movements as this will increase muscle tension and maintain the disturbance
- The uncertainty that most people experience when suffering from back pain can be reduced by providing them with sufficient knowledge (explanation to their pain, general knowledge of back pain as a commonly occurring benign condition with a good prognosis in terms of the natural healing of each episode, and knowledge on what seems to be a favorable way of self-managing the condition in terms of fast recovery)
- By providing knowledge instead advice people can draw their own conclusion instead of trusting the conclusion of others. The didactic approach is called the ‘non-directive support’ approach and was first described by Fischer et al. (1997) [36]

The topics and messages in the talks were (random order):

- LBP History: The terminology in LBP (specific versus non-specific), epidemiological history of LBP pointing to that the Western World LBP epidemic (increased disability and related costs) does not seem to be caused by a deterioration of the condition of our back (biologically) but from a failed medical approach by Modern medicine (injury-model, traditional bio-mechanical ergonomic training, increased use of picture diagnostics for non-specific LBP, beliefs that work and health are negatively linked) and from LBP having become a ‘communicable disease’
- Basic anatomy: The anatomical structures of the back with a focus on the back as the strongest structure in the body and evidence that permanent serious back injuries are mainly caused by e.g. high-intensity trauma
- Pain: The basic knowledge from modern pain models stating that pain is a personal experience that is highly influenced by beliefs, experiences, and expectations of the individual. Furthermore, information about how the brain can misinterpret signals from the body (e.g. left side arm pain when having a cardiac infarct). Also, examples of situations where pain are often ignored or easily dealt with because it is believed to be non-threatening (e.g. headache, leg cramp))—to highlight that pain is not necessarily a sign of danger/injury and that our thoughts/ideas about and attention on pain highly influences the pain experience
- Causation: The unpredictable nature of LBP, the immediate ‘good’ prognosis for most cases of LBP and theories of what has been proposed to be the main causes for initial and persistent or recurrent LBP; disc degeneration, excessive overload, temporary muscular dysfunction (the ‘functional-disturbance’-model) combined with excessive attention on pain, and unhelpful beliefs and behavior. The listeners were then provided with information on what seems to be useful ways to handle acute lumbago for most people according to the ‘functional-disturbance’-model (normal movement meaning light activity and normal movement patterns, stretching, cold/heat, massage, pain medication)). It was stressed, though, that apart from normal movement, none of the mentioned ‘treatments’ are effective at either preventing or curing LBP–they are merely potential means for temporary symptom relieving, which can be favorable to be able to resume light activity and return to normal movement patterns. Long-lasting LBP was explained to often-wise be a likely result of ongoing muscle tension–either from unnatural movements or from disc degeneration, which is highly common
- Treatment: The lack of success with clearly diagnosing non-specific LBP, the minimal effects of treatments for non-specific LBP, the risk of increasing focus on pain when seeking treatment, red flags to help identify rare serious from frequent ‘normal’ back conditions, the doubtful effects of pain medication for LBP–endorphins as a natural means for pain relief
- Ergonomics: How the theory of cracked discs due to physical loads from the 1970’s became basis for practice and regulation in the following decades, the inconsistent evidence for applying traditional bio-mechanical ergonomic interventions (lifting technique), a rationale that such techniques should not be used to avoid injury as previously believed/taught but that the techniques can be used to reduce risk of temporary pain episodes from physical overload
- Disc Herniation: Explanation of what disc herniation is, its prognosis, natural healing is the most common, most people recover within 6 months, why/when to turn to surgery, common myths about disc herniation (lifting is the main cause, disc herniation means a weakened and disabled back for life etc.), disc herniation is the result of disc degeneration, which usually happens gradually, disc degeneration is frequently occurring and somewhat age-related but genetic heritage mostly decides how quickly it develops (like grey hair and wrinkles)
- Training: Evidence on training as a means to reduce LBP; not yet fully explored, training to reduce acute pain seems non-effective according to the literature whereas it seems to be effective for some of those suffering from chronic LBP, believing that training is necessary and thereby, feeling more or less responsible for getting LBP if this is not followed is not helpful, moderate activity seems to be best for relieving LBP and that can involve all types of activities–preferably something that is perceived fun
- Coping: Characteristics in terms of beliefs and behavior of those who recover according to the empirical literature

(The slides used in the talks can be obtained by contacting the corresponding author).

At the end of the second talk, participants were offered two brief booklets: one repeating the main messages from the talks and one containing overall stretching exercises for muscle groups relevant for LBP. The latter was applied to accommodate to wishes from the participants. The exercises were not demonstrated, only presented orally and participants were not directly encouraged to do the exercises. They were told that they might experience the exercises as helpful in term of relieving pain and if so, could choose to continue doing them. It was stressed that there is no evidence supporting that stretching can neither prevent nor cure LBP. In addition to the two pamphlets, the intervention group was offered the option of calling the primary investigator (who is physiotherapist) if questions occurred subsequently during the follow-up period.

The control group did not receive any intervention by the research group, but they were offered to have the talks including booklets at study completion, which was announced at the recruitment meetings. Both groups continued to have access to usual healthcare during the study.

### Data collection

The data collection for follow-up took place between 4^th^ of March 2013 and 22^nd^ September 2014 (Fig 2). At baseline, information on demographic factors, job-related factors, lifestyle, self-rated health, LBP history, back beliefs and LBP-behavior was collected from participating workers using a questionnaire. Important baseline items and items on primary and secondary outcomes came from previously validated questionnaires: Core Outcomes Measure Index (COMI), Back Beliefs Questionnaire (BBQ), Work Ability Index (WAI), and Subjective Health Complaints Inventory (SHCI) [37–40]. For the present study, items were translated into Danish and back to English in cooperation between the primary investigator and a linguistic professional to ensure that the translation into Danish did not entail any misconceptions. Nine items from the baseline questionnaire constituted the items used in follow-up assessments (see ‘Outcomes’ section).

The 12-month follow-up period involved monthly data-collections using text messaging (SMS-tracking). Participants could use either their work or private cell phones. Five participants were offered short monthly telephone interviews because they were unaccustomed to SMS. Questions were slightly modified to fit the SMS-format and to enable answers to be given on numeric scales. Participants could respond at any time during the day. A reminder SMS, if relevant, was sent on day two. After approximately four days, non-responders were contacted by telephone. SMS-Track A/S (www.sms-track.com) was used to do the follow-up SMS messages to ensure timely, consistent data-collection, and a confidential handling of the data. Each monthly follow-up assessment was initiated by a question about the total amount of days with LBP (+/÷ sciatica) during the past month. Only those reporting LBP days in the past month were sent additional questions measuring the nine selected outcomes related to LBP behavior (see below).

### Outcomes

The outcomes were self-report items selected to reflect beliefs about, and behaviors due to, LBP. Previous studies testing information on the ‘functional-disturbance’-model have used register data on sick leave. Unfortunately, this was not an option in the present study because, at the time of the conduction of the study, a register in Denmark recording sickness absence of duration less than three weeks did not exist [41].

#### Primary outcomes

The primary outcomes were ‘cut-down days’ and ‘days of work participation’. Both are central components in pain-related behavior. The main item measuring self-restriction of activity (‘cut-down days’) was: “*Since our last data-collection*, *about how many days did you cut down on the things you normally do for more than half a day because of LBP (+/÷ sciatica)*?” Responses were registered in full days. Work participation was defined as days of attending work, and was measured using the following item: “*Since our last data-collection*, *how many (half or full) days did LBP (+/÷sciatica) keep you from going to work*?” Responses were registered in half or full working days. As data collections took place once a month, data on the primary outcomes referred to days of cutting down on activity or days of participating at work in *the past month*. Both items are part of the COMI [42].

#### Secondary outcomes

The Work Ability Index (WAI) is a self-assessment measure of perceived ability to work, and can help identify individuals who are at risk of a negative rehabilitation outcome: it is not LBP specific [43]. We used a single item-validated item from the WAI scale [44] slightly modified to suit the text format: “*On a scale from 0 to 10*, *how is your current work ability compared with your life-time best*? *(0 = no work ability—10 = optimum life-time work ability)*”.

Pain behavior is reflected in the extent of health care seeking. To measure this outcome, participants were requested to summarize total number of visits to (any) healthcare professionals (‘HCP-visits’) that they had made during the *past month* (*“Since the last data collection*.*”*).

Other secondary outcomes included were LBP-related ‘bothersomeness’ and ‘activity restrictions’ from the COMI. To be consistent with the original format of the COMI, both referred only to the workers’ experience during the *past week*: “*On a scale from 1 to 5*, *how much has your LBP(/sciatica) bothered you in the past week*?” and “*On a scale from 1 to 5*, *how much has your LBP(/sciatica) restricted your normal activity during the past week*?”. Reponses “1” equaled “not bothered/restricted me at all” whereas “5” equaled “bothered/restricted me to a maximum level” [42].

Frequency of pain medication intake was measured using the following item: “*During the past month*, *how often have you used pain medication (for LBP or any other reason)*?” Responses were four categories: “Not at any time”/”Less frequently than every week”/”On a weekly basis”/”Daily” [45]. The level of feeling sad or depressed was measured using: “*During the past month*, *to what extend have been affected by sadness/depression*?” Responses-categories were: “Not at all”/”A little”/”To some degree”/”To a high degree” [46]. Both items were included in follow-up to enable us to consider their potential confounding effects on reports of LBP behavior. The items were included at every other data collection (six times each) to reduce the total amount of items used each month from nine to eight.

Beliefs and behavior are likely to be strongly linked. The BBQ was developed to explore the beliefs in a population with or without a back pain history–equivalent to the population in the present study. We measured back beliefs using four items from the BBQ [25]:

- *“On a scale from 0 (completely disagree) to 6 (completely agree)*, *how much do you agree with the following*: *back pain means long periods off work” (item 10)*
- *”…*: *once you’ve had back trouble it will always be weakened” (item 12)*
- *”…*: *when you have back trouble you should rest” (item 13)*
- *“*:*…back trouble worsens later on in life” (item 14)*.

These items were selected due to their relevance to working life issues when dealing with back trouble. During follow-up, data on back beliefs were collected once only: at 5.5 months—between two regular monthly assessments. With monthly data collections on the behavior outcomes, we wished to reduce the number of times participants had to respond. Furthermore, we believed that if the talks had produced changes in beliefs, these changes would be profound and long-lasting because they were governed by a new basic understanding of LBP. Finally, the data collection on back beliefs was going to involve all participants and not only those reporting LBP, why it was more practical to conduct separately. Therefore, we decided to measure beliefs only once during follow-up: at 5.5 months.

### Blinding

It was not possible to blind the participants or the primary investigator (PF) during the study. However, when performing the statistical analyses the researchers (LLA/PF) was blinded to group allocation (assessor blinding). The group-allocation code was broken only after completion of the statistical analyses.

### Ethics and trial registration

The Regional Committee on Health Research Ethics in the Capital area (no. H-4-2012-129) and the Danish Data Protection Agency (no. 2012-41-0361) approved the study, which followed the principles of the Helsinki declaration of 1975 as revised in 2013 [47]. Participation in the project was voluntary, and participants could withdraw their (written) consent at any time. The intervention did not involve any risk of harm for the participants, either in the intervention or in the control group, and they were informed about this at the entry to the study. Thus, we did not expect to see any unintended effects. The study was reported to clinicaltrials.gov (reg. no. NCT01918228) in April 2013. Due to lack of awareness from the author in charge, the study was registered in clinicaltrials.gov five months into the recruitment of participants instead of before study start. The authors confirm that all ongoing and related trials for this intervention are registered. The full trial protocol can be obtained from the PLOS ONE website (Supporting Information: ‘S2 Appendix’).

### Statistical methods

#### Sample size

With no findings of any previous reports on minimal clinically important difference (MCID) on the primary outcomes items, we estimated MCID to be a 0.5 difference in mean cut-down/sick days per month and set a realistic estimate on a standard deviation of 1.3 [48]. With a power of 0.80 (two-tailed alpha = 0.05), we would need 108 persons in each group reporting LBP and providing LBP-behavior data at each monthly data-collection. In cluster randomized trials the intra-cluster correlation coefficient, if higher than 1, increases the necessary sample size [49, 50]. For the present study we had no information about the intra-cluster correlation coefficient for sick days. Assuming an intra-cluster correlation coefficient of 1.2 this would inflate the sample size to 130. With a detected 1-month prevalence of LBP (self-report) of 44–63% in populations of workers with physically demanding jobs [51, 52] and to allow for some degree of dropouts, we decided to include approximately 250 participants in each group.

#### Data preparation

At baseline, LBP-days were measured in four categories (“0”/”1–10”/”11–20”/”>20” days) and during follow-up as a continuous variable. Thus, we made estimates of the baseline means/medians in each category. Instead of estimating respective mid-range per category, we estimated weighted means for each category using the observed 12 follow-up means in the intervals 1–10, 11–20, and >20, respectively.

Because the length of the 12 follow-up ‘months’ were either four or five weeks, primarily depending on public holiday, we, subsequently, normalized all four-week numeric data to five weeks by multiplying with 1.25.

On the back-beliefs outcome, we normalized the 0–6 scale responses to a 1–5 scale in order to fit the original scale used in the BBQ. The four ratings were summarized into a total sum score (4–20). This scale was normalized to a 0–100 point scale during analysis to ease the interpretation.

#### Data analyses

Numeric data on most outcomes (baseline and follow-up variables) except back beliefs were not normally distributed. Therefore, non-parametric tests (Mann-Whitney U test) were used for the analyses of differences in background characteristics on these numeric variables.

Follow-up data on behavior outcomes were analyzed using cumulative logistic regression (Proc Genmod of SAS version 9.4). One-year-means for each of the participants (based on minimum 1 and maximum 12 responses from the monthly assessments) were used to compare groups. Group (intervention/control) was entered in the model as a fixed factor. Cluster was entered using the repeated option (repeated subject = *cluster variable*) in Proc Genmod, meaning cluster was a random factor. The analyses were adjusted for the following fixed factors: baseline value of the outcome measure and in a second set of analyses also for gender, age, smoking, and type of work (manual versus administrative).

Follow-up data on the back beliefs outcome was analyzed using linear mixed models (Proc Mixed, SAS version 9.4). This statistical procedure requires the residuals to be normally distributed: the residuals for the BBQ data were normally distributed. Group (intervention/control) was entered in the model as a fixed factor. Cluster was entered as a random factor. The analysis was adjusted for the baseline value of the outcome measure, and in a second analysis also for gender, age, smoking, and type of work.

We carried out intention-to-treat analyses (all randomized participants), per protocol (minus those not completing the intervention and those lost to follow-up), and drop-out analyses (lost to follow-up). The IBM SPSS/PASW statistics version 22 and the SAS statistics version 9.4 were used for analyses.

## Results

Fig 1 displays the flow of participants during the study. Of approximately 900 potential participants, 505 consented to participate, and 495 were eligible for follow-up and analyses (intention-to-treat). A group of participants in the intervention group did not participate in any of the talks (n = 27) and 23 persons were lost to follow-up, why the subsequent per protocol analyses included 445 participants.

The response-rates during the 12 follow-up assessments were high in both groups (84.5–94.2%) and the lowest one-month response-rate detected in one cluster in any of the groups was 70%.

The one-month prevalence of LBP at baseline in the study population was 55.6%. Of those, a total of 32.6% reported to have had cut down on usual activities for more than half a day due to LBP, and 11.0% stated that they had LBP-related days off work.

Table 1 shows the baseline characteristics of the two groups. The control group consisted of more men, more smokers and more half- or full-day manual workers. The controls also reported higher physical job demands, they scored higher on negative back beliefs, they rated their overall workability to be lower, they felt more bothered by their LBP, and the pain entailed more restrictions in their activity compared with the intervention group.

**Table 1:** Baseline characteristics on demographic factors, work-related factors, factors concerning overall health, low back paindays, and low back pain-behaviour (n = 495).

	Intervention group						Control group						*Sig*.	
N of questionnaire responders eligible for analysis	n = 256						n = 239							
Demografic factors	n	%	Mean	SD	Median	IQR	n	%	Mean	SD	Median	IQR	P ^β^^£^	
Percentage male	194	76.1	-	-	-	-	201	84.5	-	-	-	-	0.024	*
Age	255	-	49.10	10.52	51.00	44.00–57.00	236	-	48.10	10.76	50.00	42.00–55.75	0.279	
Percentage smokers	67	26.2	-	-	-	-	94	39.5	-	-	-	-	0.002	*
Percentage of unskilled workers	87	34.3	-	-	-	-	93	39.4	-	-	-	-	0.261	
Percentage with half- or full day manual work	172	67.2	-	-	-	-	218	92.0	-	-	-	-	<0.001	*
**Work-related factors**														
Self-reported physical job demands 0 (low) – 10 (high)	252	-	4.59	3.02	5.00	2.00–7.00	231	-	5.84	2.41	6.00	5.00–8.00	<0.001	*
Overall job satisfaction 0 (low) – 10 (high)	252	-	7.69	1.65	8.00	7.00–9.00	232	-	7.56	1.84	8.00	7.00–9.00	0.546	
Leasure time activity: 'mostly sedentary' or 'light activity'	175	69.2	-	-	-	-	163	70.3	-	-	-	-	0.843	
**Overall health and back pain factors**														
Percentage rating health as poor/very poor	32	12.6	-	-	-	-	26	11.1	-	-	-	-	0.675	
Self-reported days of sickness absence (all causes) during the past 12 months	224	-	7.28	10.84	5.00	2.00–10.00	205	-	7.39	10.65	5.00	1.00–10.00	0.888	
Percentage affected by illness/specific (back/other) pain conditions	69	27.7	-	-	-	-	58	25.3	-	-	-	-	0.605	
Back beliefs sum: 4–20 (20 = faulty beliefs)	250	-	11.51	3.43	11.89	9.20–13.90	235	-	12.50	3.44	12.50	10.50–14.60	0.002^€^	*
Percentage with frequent/constant LBP	82	41.4	-	-	-	-	69	37.5	-	-	-	-	0.464	
Self-rated chance of recovery from LBP within 6 weeks 0 (good) – 10 (poor)	130	-	2.18	2.70	1.00	0.00–3.00	125	-	2.49	2.66	2.00	0.00–4.00	0.238	
Paindays last month	246	-	5.64	9.04	4.34	0.00–4.34	218	-	5.57	8.27	4.34	0.00–4.34	0.267	
**Pain behaviour among those reporting LBP at baseline**														
Cutdown days last month	234	-	1.16	4.57	0.00	0.00–0.00	214	-	1.14	3.49	0.00	0.00–0.00	0.535	
Sick days last month	238	-	0.19	0.89	0.00	0.00–0.00	216	-	0.22	0.92	0.00	0.00–0.00	0.657	
Workability 0 (none) – 10 (lifetime best)	243	-	9.06	1.52	10.00	8.00–10.00	219	-	8.52	1.83	9.00	8.00–10.00	<0.001	*
HCP visits last month	240	-	0.30	1.04	0.00	0.00–0.00	212	-	0.43	1.16	0.00	0.00–0.00	0.051	
Bothersomeness last week 1 (not at all) – 5 (to a maximum level)	244	-	1.27	1.43	1.00	0.00–2.00	218	-	1.56	1.51	2.00	0.00–3.00	0.035	*
Restricted activity last week 1 (not at all) – 5 (to a maximum level)	244	-	1.04	1.25	1.00	0.00–2.00	220	-	1.32	1.33	1.00	0.00–2.00	0.019	*
Percentage with any pain medicine intake	124	51.0	-	-	-	-	124	59.0	-	-	-	-	0.090	
Percentage with some degree of reported sadness/depression	123	50.6	-	-	-	-	123	58.6	-	-	-	-	0.108	

^β^ Proportions were tested using Chi-square tests.

^£^ Medians were tested using Mann-Whitney U test.

^€^ Means (back beliefs outcome only) were tested using unpaired t-test.

*P < 0.05

Table 2 shows the results of the cumulative logistic regression analyses on the primary outcomes when adjusted for baseline, gender, age, smoking, and type of work. The two groups did not differ significantly in their reports of ‘LBP days’. Reports of ‘cut-down days’ did not differ significantly between groups at follow-up, whereas the reports of ‘work participation’ did; The intervention group had 83% higher odds for attending work during LBP episodes over the follow-up period (OR = 1.83 (95% CI:1.08–3.12)). Per protocol analyses showed similar results for the adjusted primary outcomes.

**Table 2:** Cumulative Odds Ratios (OR) for reporting fewer LBP days, fewer cut-down days, more days work participation, lower work ability, fewer visits to healthcare professionals, less use of pain medication, and lower levels of feeling sad or depressed among intervention-group participants. Control group were reference.

Variables/Estimates	C OR^$^	95% CI	C OR^#^	95% CI	*P*^₤^		*P*^†^	
**Fewer LBP days**								
Intervention group	1.50	0.91–2.49	1.34	0.85–2.13	0.209		0.188	
Control group	1		1					
**Fewer cut-down days**								
Intervention group	1.43	0.87–2.36	1.22	0.77–1.93	0.406		0.249	
Control group	1		1					
**Work participation days**								
Intervention group	2.43	1.44–4.08	1.83	1.08–3.12	0.026	*	0.016	*
Control group	1		1					
**Higher work ability**								
Intervention group	1.83	1.19–2.83	1.40	1.01–1.94	0.043	*	0.091	
Control group	1		1					
**Fewer visits to HCP**								
Intervention group	1.68	1.00–2.83	1.72	1.01–2.94	0.046	*	0.059	
Control group	1							
**Lower bothersomeness**								
Intervention group	1.44	1.02–2.05	1.50	1.06–2.11	0.022	*	0.009	*
Control group	1		1					
**Lower restricted activity**								
Intervention group	1.32	0.84–2.08	1.27	0.82–1.98	0.291		0.254	
Control group	1		1					
**Less use of pain medication**								
Intervention group	1.42	0.78–2.57	1.53	0.84–2.79	0.165		0.152	
Control group	1		1					
**Lower levels of feeling sad/depressed**								
Intervention group	1.56	0.97–2.52	1.80	1.10–2.96	0.020	*	0.019	*
Control group	1		1					

^$^ adjusted for baseline

^#^ adjusted for baseline, sex, age, smoking, type of work and cluster

^₤^ intention-to-treat analyses—adjusted for baseline, sex, age, smoking, and type of work (n = 495)

^†^ per protocol analyses—adjusted for baseline, sex, age, smoking, and type of work (n = 445)

* P<0.05

On the secondary outcomes, analyses (adjusted) showed that odds for reporting high ‘Work ability’ were generally higher among interventions group participants (OR = 1.40 (95% CI:1.01–1.94)), whereas odds for reporting no ‘Visits to HCPs’ (OR = 1.72 (95% CI:1.01–2.94)), low ‘Bothersomeness’ (1.50 (95% CI:1.06–2.11)), and low levels of ‘Feeling sad/depressed’ (OR = 1.80 (95% CI:1.10–2.96) were higher in this group compared to the controls. ‘Work ability’ and ‘Visits to HCPs were no longer significantly different when groups were analyzed per protocol.

The outcomes ‘Restricted activity’ and ‘Use of pain medication’ did not show significant between-group differences at follow-up in either of the analyses.

**Fig 3:**
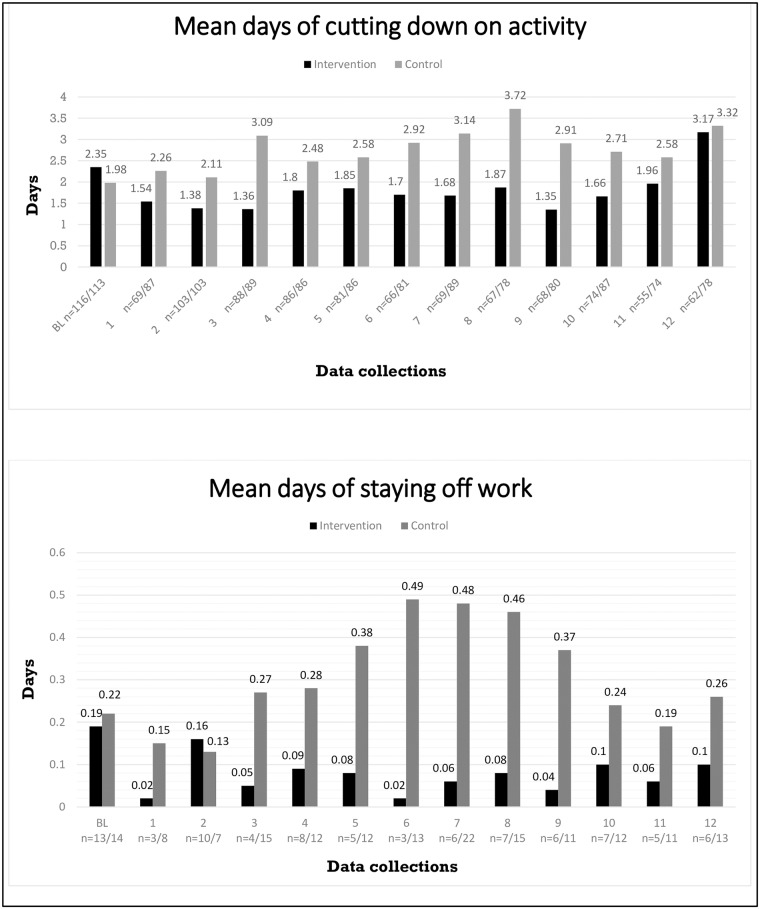
Means of LBP-related days of cutting down on activity and staying off work at baseline and at the 12 follow-up assessments for all participants. Below are the numbers of participants contributing to each mean from the intervention and control group, respectively.

Fig 3 displays the means for days of cutting down on usual activity and staying home from work due to LBP at each of the 12 follow-up assessments in the two groups. At baseline, the control group had the lowest mean on both outcomes whereas during follow-up, the intervention group reported fewer days in general.

Table 3 shows the findings for back beliefs. Linear mixed model analyses adjusting for baseline levels, sex, age, smoking, type of work showed that the intervention group scored significantly lower on a scale of unhelpful back beliefs compared with the control group at follow-up. Seen overall, the intervention group’s average sum score of unhelpful back beliefs was estimated to be 10.22 lower compared with that of the control group measured on a 0–100 point scale. Per protocol analyses showed similar results.

**Table 3:** Multiple regression analysis of back beliefs sum score^$^ in the intervention group and control group, respectively at 5.5 months follow-up. Higher score means more negative back beliefs.

	Mean	Lower-upper	Estimate	Lower-upper	*P*^£^		*P*^€^	
**Intervention group**	38.67	34.74–42.59	-10.22	-14.93—-5.50	<0.001	*	<0.001	*
**Control group**	48.88	44.47–53.30	1	-				

^$^ A 4–20 sum score normalized to a one-hundred points scale

^£^ Intention-to-treat analyses, adjusted for baseline, sex, age, smoking, and type of work

^€^ Per protocol analyses, adjusted for baseline, sex, age, smoking, and type of work

* P<0.05

During the entire follow-up period, only two participants used the offer to make contact with a physiotherapist during the study for questions regarding LBP.

There were no incidences indicating any harm from participating in the study, nor did we experience any unintended effects in any of the groups.

## Discussion

In this cluster RCT, reassuring information based on biopsychosocial principles, with a focus on the ‘functional-disturbance’-model, was delivered in two one-hour talks to several workforces. At follow-up, the groups showed no differences in the reports of the number of LBP days. However, the intervention group reported significantly more days of work participation, higher overall workability, fewer visits to healthcare professionals, lower levels of bothersomeness and reduced sadness/depression were found in the intervention group during the follow-up-year. Furthermore, the intervention group reported more positively on back beliefs compared with the controls, when measured six months after attending the talks. Some between-group baseline differences were found, which were most likely caused by the fact that the proportion of manual workers was uneven in the two groups, with higher proportions in the control group. However, we adjusted for baseline reports on most of them: gender, age, smoking, and type of work to control for their influence on the between-group comparison. Thus, it seems reasonable to attribute the between-group differences at follow-up to the intervention. Indeed, the concomitant finding that the intervention group reported more positively on back beliefs compared with the controls six months after having attended the talks, supports that conclusion.

In general terms, this trial supports those previous studies that provided evidence-informed psychosocial information and advice to groups of people, irrespective of whether they were experiencing back pain [25, 27]. The primary intentional difference in the present study was the content of the information and advice. While previous psychosocial information has specifically avoided delivering biomedical information on anatomy/physiology and avoided providing a specific biological explanation for the pain, this intervention included both, with a focus on a believable non-threatening explanation for the pain. This approach to information and advice, the ‘functional disturbance’-model, has been shown to be effective in other settings [21–24], and was apparently effective when delivered to groups of workers in educational talks. Also, this approach was seemingly was no worse than the ‘non-bio’ approach that has been used previously.

There is, though, an interesting inconsistency in the findings for the primary outcomes. While cut-down days did not differ significantly between groups, days of work participation did differ. Although the two variables seem conceptually somewhat similar, they may be measuring different things, which may represent perceptual or real differences. The message in the talks concerning activity was that light activity during an episode of LBP seems advantageous. However, the perception of ‘light’ will depend on what is the norm for a given group. If, for instance, the participants usually remain active during LBP episodes, one could hypothesize that the talks may in fact have influenced some participants to reduce their non-work activity as some sort of compensation for continuing work activities. If so, this would potentially mean that even if a proportion became more active, this would not be shown in the present results. In addition, although it could appear from the month-to-month replies (Fig 3) that the intervention group had fewer cut down days than the controls, this was not statistically significant. A previous study on the effect of information based on the ‘functional-disturbance’-model also using self-reported activity limitations as an outcome, found non-significant trend towards an positive effect of the intervention (information and one stretching exercise) on activity limitations [23].

In terms of the finding on work participation, the previous studies investigating the effect of information on the ‘functional-disturbance’ model have shown significantly positive effects on work participation [21, 22, 24]. However, the previous studies all incorporated other elements into their interventions, whereas the present study has tested the isolated effect of the information. Thus, it adds new evidence about the model and indicates that the ‘functional-disturbance’ information element may be effective. This conclusion is supported by two studies, one qualitative and one quantitative study, both investigating the influences from the information-based workplace intervention tested by Odeen et al. (2013) [24] on workers’ behavior/ability to cope with their pain. The qualitative study found that being provided with an explanation to the pain and reassurance that the pain was not a sign of danger helped workers cope with their pain and encouraged them to exceed their previous physical limits due to reduced fear [53]. The quantitative study showed that pain-related fear was a predictor of the effect shown in the study [54]. Reducing pain-related fear was mainly addressed in the information-element of the intervention.

In general, the population-based studies testing simple information-based interventions have been more effective at altering beliefs about back pain than back pain-related behavior outcomes such as work participation [25, 27, 28, 55]. Thus, it is notable that four of five studies, including the present, using the model proposed by Indahl (1999) [10] have shown consistent positive results on both beliefs and work participation, irrespective of their ‘nature’ as either clinical studies involving LBP patients or population-based studies involving workers with varying LBP status and experience [21, 22, 24]. The fifth study using the model did not measure work participation [23].

The increased work participation in the intervention group coincided with a positive influence on the secondary outcome of work ability. It seems likely that the two outcomes are related as Hagen et al. (2005) suggested, based on an intervention study including patient education, where they showed that reduced self-reported work ability was a predictor for not returning to work [56]. However, contrary to the scale used by Hagen et al. (2005), the WAI used in the present study is not condition specific. Thus, our respondents rated their overall work ability, not solely their work ability related to LBP.

Another secondary outcome that might have been influenced by the intervention was the frequency of healthcare visits. The use of health care-outcomes has not been part of the previous studies of the ‘functional-disturbance’-model, but a recent systematic review concluded that a range of information-based or education-based interventions did not produce significant reductions in the use of health care [57].

There were only slight differences between the results from the per-protocol and the ITT analyses. Thus, the p-values were either lower or higher depending on which variable were assessed. The general picture is that the results from the ITT (main analyses) and per protocol (supplementary analyses) were similar.

The item ‘Bothersomeness’ has been shown to be a valid single item for measuring pain severity [58]. The present finding on this outcome indicates that the reassuring information made participants perceive their LBP as less severe, an effect that was also significant in per protocol analyses. Two previous studies testing the effect of information-based interventions also reported positive effects on self-reported severity of symptoms [26, 59], whereas Cherkin et al. (1996) did not find effect of a similar intervention on perceived symptom reliefs [60]. Since these studies delivered somewhat different messages in different ways, perhaps the effect on pain depends partly on the nature, style and consistency of the messages, a feature that has been considered previously to be important [61].

Reporting feelings of sadness/depression was reduced by the reassuring information as well. This is something of an unexpected finding since it is difficult to see why (positive) information about back pain, delivered generically outside a healthcare setting, should influence sadness/depression. Indeed one of the previous studies involving information on the ‘non-injury’-model found no effect on depression level in patients with subacute LBP [59]. In addition, two studies have explored the effect of self-management programs on chronic pain patients and found contradictory results on the effect of the programs on self-reported depression [62, 63].

The results of the present study indicate that the actual information component might, in fact, be the more important part of brief intervention studies such as the former ones on the ‘functional-disturbance’ model [21–24]. More importantly, perhaps, it adds to the discussion on how simple an effective intervention can/should be. Previous studies have indicated that simple or brief interventions are equally effective as the more complex multidisciplinary interventions in terms of improving various clinical and work-related outcomes [64, 65]. In addition, a recent review evaluating the effectiveness and cost-effectiveness of most community- and workplace-based interventions to manage musculoskeletal-related sickness absence and job loss suggested a future focus on simple interventions [66]. There is also need to determine the long-term effects of simple interventions. It seems that there can be a long-term effect in terms of belief-change [67], but the longer-term effect on behavioral variables is less certain. Nevertheless, two of the previous studies using information on the ‘functional-disturbance’-model showed that intervention has the potential to produce longer-lasting effects on work participation [33, 34]. A third study based on the model showed that the effect was limited to six months, though, and the authors concluded that perhaps their findings indicate that when providing people with group-based information as the main intervention, the messages needs frequent repetition [54]. In the present study, analyses (12-month means) did not allow us to say anything about how long-lasting the effect might be, but Fig 3 (monthly means of the primary outcomes during the 1-year follow-up) it seems that the effect is not limited to the first six months. Another issue to consider is that of individual versus population intervention. The present study confirms that simple educational interventions can have an influence on work participation for those workers who experience LBP. However, there is a considerable literature supporting the concept of providing individual workplace support (such as transitional work arrangements) to facilitate early return to work (or work retention) for workers experiencing work-relevant back pain [68, 69]. Furthermore, in recent qualitative studies, workers themselves have suggested that such things as having adjustment latitude/leeway at the workplace improves their possibilities for coping with LBP at work [70, 71] while low ‘job control’ has been suggested as a predictor for poor occupational outcomes such as return-to-work [72]. Some of these issues are discussed in detail elsewhere [61], but for the present discussion it is, perhaps, sufficient to say that there is seemingly good reason to combine population and individual biopsychosocial educational approaches if work participation behaviors are the target outcomes.

Since 80% of the participants in our study population were manual workers with half or full day physical work, the findings are highly generalizable to a manual worker population and less generalizable to a general working population.

### Strengths and limitations

Risk of bias was markedly reduced by the high response-rates. Thus, the low drop-out rates (<5%) between baseline and follow-up would not be able to influence results notably. It seems plausible that our data collection method (SMS service combined with telephone interview), the manageable number of questions in follow-up data-collections, and the fact that the primary investigator (PF) conducted all steps of the study involving participants contributed to this. Macedo et al. (2012) showed that combining SMS service with telephone interviews improved response rates quite substantially compared to using SMS service alone and concluded that the combination is a feasible way to collect simple data [73].

The choice of a cluster-randomization brought strength to the study by reducing ‘contamination’ between intervention and control group. Furthermore, ensuring that all five municipalities were represented in both groups reduced potential bias from geographical/cultural differences between clusters.

The study also had some obvious design-related limitations. The fact that some participants may be have ‘learned’ that by reporting “0” LBP days they would not have to answer on any more questions is a potential weakness in the design of data-collection. This may have involved bias from the non-detectable proportion of people responding “0” LBP days, when they in fact had days with LBP. However, this factor would equally affect the intervention and control groups, and thus not represent a systematic bias. The randomization resulted in unequal proportions of manual and sedentary workers across the two groups; however we adjusted for these baseline differences (as well as for differences in gender, age, smoking, and type of work) in the statistical analyses. The fact that our data, particularly regarding LBP-related work participation, solely relied on self-report may have produced inaccurate reports, due, for instance, to recall bias of sickness absence days. However, it is notable that our findings on the work participation outcome were in line with those of the previous studies on the ‘functional-disturbance’-model, which all used register-based data. This could indicate that such bias was not a big issue in our data. Furthermore, due to municipalities’ data policies, we were not able to extract information on non-participating employees in order to assess if our population was representative for municipality workers in general. Another limitation is that all outcomes were self-reported and that participants could not–due to the nature of the intervention–be blinded to group allocation. Thus, placebo effects and reporting bias cannot be ruled out. Finally, the fact that we had to make a guess on the MCID used for power calculation (due to lacking evidence in the existing literature) could be regarded as a limitation as it introduced uncertainty about the precision of our selected sample size. However, as shown in Table 1, the SD was 1.2, whereas we had estimated it to be 1.3. Using an SD of 1.2 in a post hoc statistical power calculation the required sample size of each group would be 91 instead of 107. This indicates that we seemingly did include sufficient numbers of participants for the two groups.

## Conclusion

The findings from the present study, and from a similar previous study using educational information on the “functional-disturbance”-model, indicate that providing workers with this kind of information, whether or not they are experiencing back pain, can produce significant alterations in both beliefs and behavior during subsequent back pain episodes. This is analogous to the similar results when the model is delivered to patients in a clinical setting. Future studies should focus on: testing how thorough the information should be; how the long-term effect is; and if the information-based interventions should be added to other relevant elements to be more effective. The results add to the discussion of how little is best when designing interventions with the purpose of reducing the risk of pain-related disability and work loss.

## Supporting information

S1 Appendix S1 CONSORT checklist. (DOC)

S2 Appendix S2 Study_protocol_v24082012_ENGLISH. (PDF)

S3 Appendix S3 Study_protocol_v24082012_ENGLISH. (DOC)

S4 Appendix S4 Corrections study protocol June 2016. (DOCX)
