# Supplementary material for: Can group-based reassuring information alter low back pain behavior? A cluster-randomized controlled trial
Source: PLoS One. 2017 Mar 27;12(3):e0172003. doi: 10.1371/journal.pone.0172003 (PMC5367686; doi:10.1371/journal.pone.0172003)
Supplement: S3 Appendix — (DOC) [file pone.0172003.s003.doc]

**Confidence in the back**

**– and in the work**

A randomized controlled observer-blinded trial

by

Pernille Frederiksen¹, Tom Bendix^1^, Anne Keller^1^ og Aage Indahl²,³

1 Videncenter for Reumatologi & Rygsygdomme, Glostrup Hospital

2 Sykehuset i Vestfold, Klinikk fysikalsk medisin og rehabilitering, Stavern, Norge

3 Rikshospitalet, Oslo, Norge

The present study has been planned as a PhD project with Tom Bendix Sc.D as head supervisor, Anne Keller PhD as project supervisor and Aage Indahl PhD as co-supervisor.

It will take place at Copenhagen Spine Center, Glostrup University Hospital, Denmark and at a number of municipal Technical end Environmental Departments in the Capital Region.

24.08.2012

**Introduction**

Back pain (BP) and its effects on the daily functioning have certainly always exsisted.^1-3^ A Danish study by Biering-Sorensen et al. (1978)^4^ found a 1-year prevalence of BP of approximately 50% in the general population, which does not seem to have increased since then, ^5,6^ – underlining that pain mechanisms hardly has changed substantially biologically speaking.

The present back problem has been added an extra dimension in the form of back-related sickness absence. Pain-related sickness absence increased dramatically from late 70s to mid-90s and after that continued to stay at a high level.^2^ The problem appears to be biggest in public sector workplaces.^7^

Annually, BP cost the Danish Society 17 billion. Of that, 7.3 billion is due to productivity losses, and 5.6 billion is due to treatment expenses.^8^ Apparently, the increase in the absence is related to two components: 1) access to worker’s compensation and 2) fear avoidance beliefs/behavior – whereas occupational factors such as work load are less influential.^9^

Cf 1: Benefit/compensation is important on two levels: The increase in absenteeism ^10-12^ - the mechanism is most clearly illustrated this in the former East Germany, where BP-related absenteeism were almost entirely absent before the reunion of Germany, but rose to Western levels after the reunion, as economic benefits became available. ^13^ This is supported by studies comparing outcomes of work-related back injuries versus leisure-time-related back injuries, which have shown worse outcomes (eg. pain ratings) in people with work-related injuries, who have access to compensation. ^10,11^ Moreover, in countries where Modern Medicine at the time had not yet become predominant, there was no similar tendencies in sick leave rates although the BP frequencies where at a similar level to the Western levels.^3^

Cf 2: Evidence points to that patients' uncertainty of what the back can tolerate (movements/loads) and their fear-avoidance beliefs and -behavior can be very important for physical functioning and work participation.^14-19^ It seems likely that the traditional bio-mechanically oriented messages pre-dominant during the 1970’s and 1980’s based on the idea of need for caution during lifting/handling loads have contributed to peoples’ fearful behavior. It was then believed that stressful loading on the spine was the main cause of the degeneration of discs (DD), which plays an important role in the initiation of BP. A high quality twin-study from 1995 proved this to be wrong. Physical loading only contributed to DD with a few percent, as opposed to genetic factors throughout the lumbar spine contributed approximately 50 %^9^ Nonetheless, the biomechanical messages involving a fundamental distrust of the capability of the back, tend to cling to the beliefs and behavior of patients and also to healthcare professionals. Studies have shown that negative beliefs about the strength/resistibility of the and avoidant behavior are related to the course of BP and the explanation to this linkage has been explained by sensitization and more tense movements patterns.^20-23^

The high rates of BP-related disability is also interesting in the light of the fact that work load, which have taken much of the blame, generally have become less strenuous with increasing focus in work regulations. Reports of BP and physical loading are associated,^24^ although the literature on it is somewhat inconsistent.^25^ However, if people have been taught that workloads inflict both BP and potentially injury, they would probably tend to attribute their pain episodes to workloads. Based on the lacking evidence of loads inflicting injuries such as disc degeneration^9^ and the evidence showing that tense movement patterns do cause BP ^26, 27^ we need to take that into account in our pedagogical approach to the patients.

Interventions aimed at the return to work can be divided into primary or secondary / tertiary prophylactic measures. Whereas the primary interventions target people at risk of BP, the second/tertiary interventions are aimed at reducing disability in people with BP.28 Examples of primary interventions designed to prevent BP are the classic person-oriented bio-mechanical ergonomic interventions, often tested at the workplace arena. Results on such have shown that such interventions are ineffective at preventing BP and even can increase BP injury reports.^26, 27, 29^ This could raise hypotheses that such interventions increase the negative beliefs and behavior related to BP.

Primary interventions that do seem to be favorable for reducing BP and related disability are better organization of the work tasks reducing the repetitive movements and cumulative workload ^30, 31^ and physical training at the workplace ^32^ Other types of primary interventions include media campaigns. Studies have predominantly reported a positive effects on the attitudes towards backache in the general population, ^33-36^ however, the impact has not been visible permanently on sick-leave statistics or other work faktorer.^34-36^

Within secondary rehabilitation interventions vary from the expensive 3-week full-time 'Functional Restoration' of the former back schools with training in lifting and other working techniques, to highly simple interventions involving a few hours of patient education. Effects of Functional Restoration interventions on sick leave have been modest^37, 38^ whereas the simple interventions have shown mixed results. A secondary intervention model, which did show effect on absenteeism and quality of life in workers experiencing BP was the Sheerbroke-model.^39^ This model involved a 3-arm intervention including a work-intervention, a clinical intervention and a combination of the two with the usual steps. It was found that workplace intervention accounted for the majority of the observed effect on sickness absence, but the effect on pain and functional level was moderate to low. At 1 year follow-up the effects had diminished, though. The study has since then include been criticized for inadequate description of metode.^40^ Also different psychologist-controlled cognitive interventions have shown some effect.^41^

The most convincing effects in the category of simple secondary interventions was seen in a study by Indahl and colleagues (1995) testing the effect a of reassuring approach to BP patients.^42^ The study did not only show immediate effect on sickness absence, it also demonstrated effect on absenteeism five years efter.^43^ It has been repeated in another context with not quite as big, but still significant effect.^44^ Both studies compared the intervention with ‘usual care’. Recently, another study has compared a reassuring approach to contemporary optimized physical training, where an average of 3 hours of reassuring information proved at least as effective as 7 sessions with physical træning.^19^ A Danish study form 2010^45^ with similar information and simultaneously call for self-selected exercise 3 x 45 min / week, also showed effect, but only 3-month data was available. In this study, they added workplace visits in 25% of workers in the intervention group with the aim of reorganizing the work of the individual if necessary.

Based on the success of Indahls’ reassuring and simple approach (1995),^42, 43^ the same research team has created a newer intervention-model: ‘iBedrift’. This model, which is mainly based on reassuring information, contained 3 elements:

1) reassuring information given as lectures to employees

2) use of a peer advisor that provides support and contact for those workers who experience BP

3) specialized healthcare offering further examination to workers with persistent problems

The elements were combined into 3 arms: one arm combining element 1 and 2 (‘A’), one arm combining all 3 elements (‘B’) and an untreated control arm (‘C’).

A long-term study is at the moment examining the effect of the two arms compared to the control arm. A large population of publically employed workers at nursing homes or in children day care institutions is included. The preliminary experiences are that only few workers use the peer advisors and the specialized healthcare option. Also preliminary results point to an effect on sickness absence and back beliefs. Therefore, it seems highly relevant to test the isolated effect of the information-based component. The content of the information is very much in line with the messages from the successful 1995-study by Indahl,^42^ however, a key difference will be the way that the information is delivered. In the 1995-study Indahl had personal conversations with each intervention-group participant, whereas in the ongoing study, the information is delivered in group-based lectures. This is an even simpler way making it possible to give information to big amount of people at once. Other studies have supported the rationale for similar group-based relying on information only. In these studies the information has been given in the form of small books or pamphlets.^46-48^ They have not managed to produce similar effects to those of Indahl. Presumably, it is due to differences in the content and form of the information that the effect of these interventions on e.g. sickness absence has been smaller and less persistent.

The purpose of this study is to investigate the isolated effect of reassuring information - inspired by Indahls method - to determine the effects on BP-related beliefs- and behavior-parameters (mainly physical function and sickness absence) among publically employed workers, who will be followed 1 year after the intervention. The focus of the study will be on low back pain (LBP), which is the main type of BP.

Our hypothesis is that the intervention will result in workers experiencing less pain-related influence on their physical functioning, less need for days of sickness absence, higher reports on work ability, fewer visits to healthcare professionals and lower reports on unhelpful back beliefs.

**MATERIAL AND METHODS**

**Design**

Controlled observer-blinded study with randomization of workplaces characterized by a moderate to high levels of physically strenuous work.

**Participants**

Potential participants will be recruited using written information to the each individual worker. The Potential participants report back to their local supervisor if they are interested in paticipating in a subsequent recruitment meeting. This meeting will take place at the workplace and the potential participant will be informed about his/her right to bring a lay representative to the meeting and of the offer to receive personal information upon request. The meeting will involve all relevant information about the study. At the end of it, a baseline questionnaire and a consent form will be handed out in an envelope with address and postage on with a kind request to decide, fill out, sign (if deciding to participate) and send back to the research team within a couple of days.

**Inclusion**

All skilled and unskilled workers employed at Technical end Environmental Departments in Municipalities in the Capital Region - approximately 400 in total.

**Non-inclusion / exclusion**

- Known/specific back disease: Mb. Bechterew, Psoriac Arthritis, Mb. Reiter. (Disc Herniantion OK). Sequelae from back surgery.
- Pregnancy within the first 9 months of the study.
- Other illness in the musculoskeletal system or organ(s) significantly affecting the person in terms of work ability (Rheumatoid arthritis, illness in the connective tissues, psychiatric conditions, other)*
- If a person has made pre-arrangements to leave the workplace/go on leave within the course of the study and if someone unexpectedly leaves the workplace/go on leave during the study

*If information on this in the baseline questionnaire, the person will be contacted. Also, if a participant during the course of the study has been absent from work ≥ 3 weeks and has not reported any back pain-related absence.

**Side effects, risks and disadvantages**

It can be considered a disadvantage for the participants that the study course is more than 1-year long involving that they will be contacted on a monthly basis and asked to give reports. In terms of side effects and risks there are apparently no identifiable.

**The intervention**

We are going to compare 2 groups: an intervention and an untreated (by us) control group each of approximately 200 workers (totally 3-5 municipal centers within the Technical and Environmental area). We will follow the two groups for 12 months.

The intervention takes place at the workplace and all participants in this group, participates in a 1-hour talk with reassuring information as described below. A follow up talk containing more (new) information will take place approximately 2 weeks after the first one. The information will be repeated in written material, which, together with the option of advice from a physiotherapist (agony column) will be available on a website right after the first talk and during the rest of the study.

The content of the talks/the written material will be:

- Low back pain historically focusing on the evidence pointing to the increasing negative consequences of BP is caused by e.g. taught caution up through the 1970’s and 1980’s, economic and cultural aspects/trends
- The anatomical structures in the back and a status on what we know about biological pain mechanisms: that crack in the discs can produce pain if they reach the disc surface and that muscle tension is produced in addition. Other topics will be the naturally unpredictable course of back pain and evidence showing that various types of treatment do not seem to work. Also, that focusing on pain do seem to influence the course of pain, -feeling insecure about what the pain is ’saying’, -believing that the back can be damaged from lifting heavy loads, that days of sick leave can be ’necessary’ but that such tend to increase pain and delay natural healing etc.
- The prognosis of back pain: back pain episodes often involve future back pain. However, many people, also among those having experienced severe back pain, pain episodes mainly take place in a certain period in their life. Later, they are typically reduced or will cease.
- The strength and capacity of the back: The biological structures are strong and will not be seriously injured from everyday loads e.g. heavy workloads. Workloads can produce pain however, as tissues can tolerate this there is no biological good arguments for restrictions.
- Red Flags – how to distinguish rare, serious back conditions from frequent non-serious back conditions
- Pain physiology: although the pain might be initiated by peripheral stimuli the brain is where it is registered. Feeling insecure/uncertain and muscle tension can both reinforce or reduce the perception of pain – like a radio volume button
- Myths and facts

The talks have the purpose of informing the participants to a degree which allows them to cope with present and future back pain episodes in a way that involves feeling reassured to e.g. maintain a normal activity level. For the talks we will use a specific pedagogic principal named the ’non-directive approach’. This approach involves that the informer avoids taking the role as advisor unless the participants ask specifically for advice. The approach has proved useful to significantly reduce body weight, blood glucose levels, and metabolic syndrome in Pakistani women with short education.^49^ The principle was also used in Indahls 1995-study^42^, which resulted in significantly more intervention-group workers returning to work compared with control-group workers. In this study the information was based on the message: ‘The back is strong’. The participants were then to decide how to act upon this information in terms of returning to work or not.

In the present study, it is important the participants will have support from the supervisors/senior management to navigate in their coping with back pain with the purpose of staying at work during pain episodes or returning to work fast. Moreover, both groups will have liberty to seek treatment/help from private and public healthcare professionals as usual (as non-participants have).

**Effect parameters**

All participants will be filling out a baseline questionnaire to uncover background characteristics (socio-demographic factors, lifestyle, self-rated health, job satisfaction etc.), beliefs about back pain (Back Beliefs Questionnaire (BBQ) item 10, 12-14^50^) and back pain history. Some will, according to epidemiologic sources reporting a point prevalence of 15-30 % within a 2-week period^6, 51^, have present back pain at baseline. Those with present back pain will also be asked to respond to item 1, 2, 4 og 5 from Low back Pain Botherssomeness scale (LBP-BS),^52^ an item on work ability (item 1 Work Ability Index (WAI),^53^ use of pain medication and number of healthcare visits. Approximately 4 months after baseline, the assessment on back beliefs (BBQ item 10,12-14) will be repeated separately.

Primary outcomes are:

- Functional level (item 2 and 4 in LBP-BS)
- Self-rated work ability (item 1 in WAI)

Secundary outcomes are:

- Sickness absence; full days (due to back pain: item 5 in LBP-BS)/general sickness absence: local register
- Back pain/-bothersomeness (item 1a+b in LBP-BS)
- Number of visits to physiotherapists, chiropractor or similar
- Back beliefs (BBQ item 10, 12-14)

Furthermore, pain medicine intake will be registered.

LBP-BS^52^ was developed with the purpose of constructing a short and relevant set of items for use in clinical back research. The item concerning work ability from the WAI is part of a larger scale but has been validated as a single item^54^ and has been used in Danish work environment-surveys in a translated version (Danish).^55^ BBQ^50^ has been developed to explore beliefs about back pain irrespective of previous back pain experience which makes this questionnaire different from most other questionnaires exploring pain-related beliefs. Neither the LBP-BS nor the BBQ exist in Danish versions why we need to translate our selected items. To do that, we will involve professional translators and the creators of the original scales. Both scales have been tested for validity and reliability,^50, 56^ but the single items in them have not. If possible, we will conduct this single-item validation concurrently with or after completion the study.

Both the baseline and the follow-up assessments will contain as few questions as possible to increase chance of good response-rates. Therefore, follow-up assessments use single items on all variables except for the primary outcome functional level which will be assessed using two items. The Norwegian ’iBedrift’-intervention-model, researchers generally used entire scales and thus, longer questionnaires and they have reported that they are obtaining rather low response-rates. We believe that the short assessments planned for the present study are adequate to obtain the relevant information. Furthermore, the longer scales would be inappropriate for the chosen method.

Follow-up assessments will take place once a month using Short Message Service (SMS). All participants receive a question on whether or not they have had back pain during the 4 (/5) weeks. If ’yes’, they will receive 7 more questions covering the primary and secondary outcomes.

If the participant has not responded within 4 days, he/she will be contacted by a (for the study) trained assistant offering the opportunity to respond over the phone. Prior to each monthly assessment, the participants will be reminded that a new assessment is starting.

**Statistics**

The demographic data will be presented both non-parametrically and parametrically – the latter to make it possible to use the results in future meta-analyses. We expect a follow-up course as illustrated in Figure 1, where half of the participants will experience back pain episodes of varying frequency, length, and intensity.

Figure 1. Expected schematic distribution of

back pain courses (blue) in both the

intervention- and the control-group

We plan to compare the intervention- and control-group using regression model such as linear mixed-model. The analyses will involve model with repeated measurements on the parameters: functional level, sickness absence and back pain, because we thereby will be able to highlight how frequently we see short and long back pain episodes with related behavior. Other parameters will be analyzed together. We plan to conduct a sort of ‘intention-to-treat’-analyses by involving ‘the last observation carried forward’. The notion ’Minimal Important Change’ will be involved and the share with the largest experienced improvement will be compared with the share with the least improvement – using logistic regression analyses. For statistical analyses SPSS will be the used software and the primary researcher plan to attend courses in regression-analyses.

**Power**

We expect that half of the participants will experience at least one back pain episode during the follow-up period. By including approximately 200 in each group we assume that half: 100+100 will report back pain, which on the basis of a power calculation^57^ should involve adequate power. We have made a realistic estimate on the standard deviation of the functional level-parameter (item 4 LBP-BS) from data in a recently published article. ^58^ With a standard deviation of 1.2, a wish of a MIC on 0.5, and a power on 0.8 (two-tailed alpha=0.05), we will need 92 participants in each of the two groups. Thus, we are going to recruit minimum 100 participants for each group.

**Blinding**

Baseline questionnaires will be handled solely the research secretary during the entire process until data is gathered for a creating a database. The task of entering the data into a database will be performed by the blinded primary investigator. The questionnaires have been anonymised using study ID making it impossible to identify the participant, his/her Work Place and thereby also group allocation. The SMS-responses will be handled by the software supplier, who will eventually deliver the gathered data in an Excell-file. The research secretary will then transfer data to SPSS in which the two groups will be named ’x’ and ’y’. The analyses will be performed by the blinded primary investigator and not until the analyses are terminated will the codes be ‘broken’.

**Aspects influencing the chance of successful completion of the study**

Experiences from Norway shows, that, in general, the work places are positive about such kind of study and that they appreciate the effort to try to help their employees with back pain – e.g. to maintain the productivity.

The control group will be offered to get the same information (1 longer talk and written material) as the intervention group received – when the study is completed. How extensive the information will be relies on the results of the study.

In the Norwegian study (somewhat similar to the present), the researchers experience problems with low response-rates. Therefore we have decided to use monthly SMS-assessments to collect responses on a few items concerning only the most important parameters and only hand out longer questionnaires once: at baseline. We add telephone interviews to the data collection to try to collect data from otherwise missing responses. Telephone-interviews generally improve the response-rates.

We have been informed that many employees at the Technical and Environmental Department in (Municipality of Copenhagen) are provided with mobile devices from the work place. Alternatively, the study participants will be able to use private mobile devices and will have any related expenses paid by the study. Participants with no access to or habit of using mobile devices (SMS) will be offered monthly telephone interviews instead.

Our close cooperation with the Norwegian research team performing a bigger however somewhat similar study has given us the opportunity to learn from them in our planning phase. This increases the possibility of successfully completing the study and for making appropriate moderations to our intervention-model. We will continue to use their experiences during the various phases of the study.

**Ethical considerations**

Testing information-based interventions is according to the empirical literature highly relevant and such interventions seem to be equally effective at preventing pain-related problems as traditional treatments.

It seems reasonable to handle ordinary back pain with straightforward methods such as reducing work load during a pain episode. Serious pathology is rare among back pain cases but our participants continue to have the option to seek professional assistance as usual if they feel they need to.

We will collect signed informed consent-forms from all participants. The information given prior to study start will have a form that does not reveal all details to the control group in order not to compromise the study.

**Economical consideration**

The main researchers behind the study are employed by Glostrup Hospital who pays their monthly salary. Conducting research is part of the tasks agreed with the work place. The additional costs related to conducting the present study will be covered by funding from the Danish Rheumatoism Association granted in the spring of 2012. At the moment there are no other funding for the study.

**Announcement of the study results**

This will mainly be done in a scientific journal but will also be done through media and lectures.

**Patents/ownership**

Not relevant for the present study.

**Reference List**

1. Bendix T, Bendix A. Alment om tackling af rygproblemet. Månedsskrift for Praktisk Lægegerning 70(6), 525

534. 1992.

1. Waddell G. The Back Pain Revolution. 1999. Churchill Livingstone, New York.
2. Wilhelmsen I, Mulindi S, Sankok D et al. Subjective health complaints are more prevalent in Maasais than in

Norwegians. *Nord.J.Psychiatry* 2007;61:304-9.

1. Biering-Sorensen F. Low back trouble in a general population of 30-, 40-, 50-, and 60-year-old men and

women. Study design, representativeness and basic results. *Dan.Med.Bull.* 1982;29:289-99.

1. Leboeuf-Yde C, Nielsen J, Kyvik KO et al. Pain in the lumbar, thoracic or cervical regions: do age and gender

matter? A population-based study of 34,902 Danish twins 20-71 years of age. *BMC Musculoskelet Disord*

2009;10:39.

1. Leboeuf-Yde C, Klougart N, Lauritzen T. How common is low back pain in the Nordic population? Data from a

recent study on a middle-aged general Danish population and four surveys previously conducted in the Nordic

countries. *Spine (Phila Pa 1976.)* 1996;21:1518-25.

1. <http://www.dst.dk/pukora/epub/Nyt/2011/NR625.pdf>
2. Koch MB, Davidsen M, Juel K. De samfundsmæssige omkostninger ved rygsygdomme og rygsmerter i

Danmark. 1-5-2011. SIF, DK.

1. Battie MC, Videman T, Gibbons LE et al. 1995 Volvo Award in clinical sciences. Determinants of lumbar disc

degeneration. A study relating lifetime exposures and magnetic resonance imaging findings in identical twins.

*Spine (Phila Pa 1976.)* 1995;20:2601-12.

1. Greenough CG, Fraser RD. The effects of compensation on recovery from low-back injury. *Spine (Phila Pa*

*1976.)* 1989;14:947-55.

1. Rohling ML, Binder LM, Langhinrichsen-Rohling J. Money matters: A meta-analytic review of the association between financial compensation and the experience and treatment of chronic pain. *Health Psychol.* 1995;14:537-47.
2. Steenstra IA, Verbeek JH, Heymans MW et al. Prognostic factors for duration of sick leave in patients sick listed with acute low back pain: a systematic review of the literature. *Occup.Environ.Med.* 2005;62:851-60.
3. Raspe H, Hueppe A, Neuhauser H. Back pain, a communicable disease? *Int.J.Epidemiol.* 2008;37:69-74.
4. Dawson AP, Schluter PJ, Hodges PW et al. Fear of movement, passive coping, manual handling, and severe or radiating pain increase the likelihood of sick leave due to low back pain. *Pain* 2011.Jul;152(7):1517-24
5. Kovacs FM, Muriel A, Castillo Sanchez MD et al. Fear avoidance beliefs influence duration of sick leave in Spanish low back pain patients. *Spine (Phila Pa 1976.)* 2007;32:1761-6.
6. Morris J, Watson PJ. Investigating decisions to absent from work with low back pain: a study combining patient and GP factors. *Eur.J.Pain* 2011;15:278-85.
7. Rainville J, Smeets RJ, Bendix T et al. Fear-avoidance beliefs and pain avoidance in low back pain--translating research into clinical practice. *Spine J.* 2011;11:895-903.
8. Reme SE, Hagen EM, Eriksen HR. Expectations, perceptions, and physiotherapy predict prolonged sick leave in subacute low back pain. *BMC.Musculoskelet.Disord.* 2009;10:139.
9. Sorensen PH, Bendix T, Manniche C et al. An educational approach based on a non-injury model compared with individual symptom-based physical training in chronic LBP. A pragmatic, randomised trial with a one-year follow-up. *BMC.Musculoskelet.Disord.* 2010;11:212.
10. Jensen OK, Nielsen CV, Stengaard-Pedersen K. Low back pain may be caused by disturbed pain regulation: a cross-sectional study in low back pain patients using tender point examination. *Eur.J.Pain* 2010;14:514-22.
11. Hodges PW, Moseley GL, Gabrielsson A et al. Experimental muscle pain changes feedforward postural responses of the trunk muscles. *Exp Brain Res* 2003;151:262-71.
12. MacDonald D, Moseley GL, Hodges PW. Why do some patients keep hurting their back? Evidence of ongoing back muscle dysfunction during remission from recurrent back pain. *Pain* 2009;142:183-8.
13. Moseley GL, Hodges PW. Are the changes in postural control associated with low back pain caused by pain interference? *Clin.J.Pain* 2005;21:323-9.
14. Hartvigsen J, Leboeuf-Yde C, Lings S et al. Is sitting-while-at-work associated with low back pain? A systematic, critical literature review. *Scand.J.Public Health* 2000;28:230-9.
15. Bakker EW, Verhagen AP, van TE et al. Spinal mechanical load as a risk factor for low back pain: a systematic review of prospective cohort studies. *Spine (Phila Pa 1976.)* 2009;34:E281-E293.
16. Daltroy LH, Iversen MD, Larson MG et al. A controlled trial of an educational program to prevent low back injuries. *N.Engl.J.Med.* 1997;337:322-8.
17. Hartvigsen J, Lauritzen S, Lings S et al. Intensive education combined with low tech ergonomic intervention does not prevent low back pain in nurses. *Occup.Environ.Med* 2005;62:13-7.
18. Zwisler A, Schou L. Hjerterehabilitering. Hjerterehabiliteringen, Kardiologisk klinik Y, H:S Bispebjerg Hospital . 10-11-2011. 10-11-2011.
19. Burton AK, Balague F, Cardon G et al. Chapter 2. European guidelines for prevention in low back pain : November 2004. *Eur.Spine J.* 2006;15 Suppl 2:S136-S168.
20. Westgaard RH, Winkel J. Occupational musculoskeletal and mental health: Significance of rationalization and opportunities to create sustainable production systems - A systematic review. *Appl.Ergon.* 2011;42:261-96.
21. Shaw W, Hong QN, Pransky G et al. A literature review describing the role of return-to-work coordinators in trial programs and interventions designed to prevent workplace disability. *J.Occup.Rehabil.* 2008;18:2-15.
22. Pedersen MT, Blangsted AK, Andersen LL et al. The effect of worksite physical activity intervention on physical capacity, health, and productivity: a 1-year randomized controlled trial. *J.Occup.Environ.Med.* 2009;51:759-70.
23. Buchbinder R, Jolley D. Effects of a media campaign on back beliefs is sustained 3 years after its cessation. *Spine (Phila Pa 1976.)* 2005;30:1323-30.
24. Gross DP, Russell AS, Ferrari R et al. Evaluation of a Canadian back pain mass media campaign. *Spine (Phila Pa 1976.)* 2010;35:906-13.
25. Waddell G, O'Connor M, Boorman S et al. Working Backs Scotland: a public and professional health education campaign for back pain. *Spine (Phila Pa 1976.)* 2007;32:2139-43.
26. Werner EL, Ihlebaek C, Laerum E et al. Low back pain media campaign: no effect on sickness behaviour. *Patient.Educ.Couns.* 2008;71:198-203.
27. Bendix AE, Bendix T, Haestrup C et al. A prospective, randomized 5-year follow-up study of functional restoration in chronic low back pain patients. *Eur.Spine J.* 1998;7:111-9.
28. Guzman J, Esmail R, Karjalainen K et al. Multidisciplinary bio-psycho-social rehabilitation for chronic low back pain. *Cochrane.Database.Syst.Rev.* 2002;CD000963.
29. Loisel P, Abenhaim L, Durand P et al. A population-based, randomized clinical trial on back pain management. *Spine (Phila Pa 1976.)* 1997;22:2911-8.
30. Karjalainen K, Malmivaara A, van TM et al. Multidisciplinary biopsychosocial rehabilitation for subacute low back pain among working age adults. *Cochrane.Database.Syst.Rev.* 2003;CD002193.
31. Vlaeyen JW, Morley S. Cognitive-behavioral treatments for chronic pain: what works for whom?*Clin J Pain* 2005;21:1-8.
32. Indahl A, Velund L, Reikeraas O. Good prognosis for low back pain when left untampered. A randomized clinical trial. *Spine (Phila Pa 1976.)* 1995;20:473-7.
33. Indahl A, Haldorsen EH, Holm S et al. Five-year follow-up study of a controlled clinical trial using light mobilization and an informative approach to low back pain. *Spine (Phila Pa 1976.)* 1998;23:2625-30.
34. Hagen EM, Eriksen HR, Ursin H. Does early intervention with a light mobilization program reduce long-term sick leave for low back pain? *Spine (Phila Pa 1976.)* 2000;25:1973-6.
35. Jensen LD, Maribo T, Schiottz-Christensen B et al. Counselling low-back-pain patients in secondary healthcare: a randomised trial addressing experienced workplace barriers and physical activity. *Occup.Environ.Med.* 2011.
36. Coudeyre E, Tubach F, Rannou F et al. Effect of a simple information booklet on pain persistence after an acute episode of low back pain: a non-randomized trial in a primary care setting. *PLoS.One.* 2007;2:e706.
37. Frost P, Haahr JP, Andersen JH. Reduction of pain-related disability in working populations: a randomized intervention study of the effects of an educational booklet addressing psychosocial risk factors and screening workplaces for physical health hazards. *Spine (Phila Pa 1976.)* 2007;32:1949-54.
38. Symonds TL, Burton AK, Tillotson KM et al. Absence resulting from low back trouble can be reduced by psychosocial intervention at the work place. *Spine (Phila Pa 1976.)* 1995;20:2738-45.
39. Johansen KS, Bjorge B, Hjellset VT et al. Changes in food habits and motivation for healthy eating among Pakistani women living in Norway: results from the InnvaDiab-DEPLAN study. *Public Health Nutr.* 2010;13:858-67.
40. Symonds TL, Burton KA, Tillotson KM and Main CJ. Does attitutes and beliefs influence workloss due to low back trouble? *Occup. Med.* Vol. 46(1)1996; 25-32
41. Kjøller M, Juel K, Kamper-Jørgensen F. Folkesundhedsrapporten 2007. 2007. SIF, DK.
42. Deyo RA, Battie M, Beurskens AJHM et al. Outcome Measures for Low Back Pain Research: A Proposal for Standardized Use. Spine (Phila Pa 1976). 1998 Sep 15;23(18):2003-13.
43. Ilmarinen J, Lehtiene S, editors. Past, present and future work ability: proceedings of the 1^st^ international Symposium on Workability, 5-6 september 2001, Tampere, Finland. Helsinki: Finish Institute of Occupational Health; 2004. People and Work, Research Reports 65
44. Ahlström L, Grimby-Ekman A, Hagberg M, Dellve L. The work ability index and single-item question: associations with sick leave, symptoms, and health – a prospective study of women on long-term sick leave. Scand J Work Environ Health. 2010 Sep;36(5):404-12
45. <http://www.arbejdsmiljoforskning.dk/da/arbejdsmiljoedata/arbejdsmiljo-og-helbred>
46. Ferrer M, Pellisé F, Escudero O, Alvarez L, Alonso J, Deyo RA. Validation of a Minimum Outcomes Core Set in the Evaluation of Patients With Back Pain. Spine 2006;31(12); 1372-9
47. [http://homepage.stat.uiowa.edu/~rlenth/Power/](https://mail/owa/redir.aspx?C=dTdOwFqxeU-KbFFeHfHTDYpxqqpjVc8IRLprggisGOlEtXhgxo6l0W6Vu1MD7K7uRh7RWk5jTQ0.&URL=http%3A%2F%2Fhomepage.stat.uiowa.edu%2F~rlenth%2FPower%2F)
48. Storheim K, Brox JI, Løchting I, Werner EL, Grotle M. Cross-cultural adaptation and validation of the Norwegian version of the Core Outcome Measures Index for low back pain. Eur Spine J. 2012; 21: 2539–49
