# Supplementary material for: Can group-based reassuring information alter low back pain behavior? A cluster-randomized controlled trial
Source: PLoS One. 2017 Mar 27;12(3):e0172003. doi: 10.1371/journal.pone.0172003 (PMC5367686; doi:10.1371/journal.pone.0172003)
Supplement: S4 Appendix — (DOCX) [file pone.0172003.s004.docx]

**Corrections/alterations made in the study after the original version (version 1_24082012) of the study protocol:**

Page 1

Title:

-The title of the protocol was only used for funding purposes and to name the study as did not reflect the title of the subsequent manuscript.

Research team/authors:

-The research team changed from including four members to begin with to include six members at the end. Anne Keller left the study before study recruitment began due to ongoing disagreements in the research team. She is mentioned in the Acknowledgements section.

-Lars L. Andersen and Kim Burton joined the study prior to commencing the analyses. Kim had already been part of the study in the planning process as he is one of the creators of the Back Beliefs Questionnaire used in the study. Kim joined because he is a huge resource within the field of back pain and work rehabilitation and because he is native English, why he could help with the language and grammar issues. Lars Andersen joined upon our request because we realized that we were not able to conduct the analyses as initially planned and needed professional statistical assistance. Furthermore, he is a huge resource on the field of musculoskeletal pain conditions as Professor for the National Work Environment Research Center.

Location for the study:

-As the recruitment proceeded, we needed to expand the search for potential participating municipalities to include the Region of Zealand as well.

Page 2-3

-Introduction: no changes

Page 4

Material & methods - design:

-We realized that our plan to conduct the study as an observer-blinded study was not possible for practical reasons. Instead, we decided that we would make sure that analyses were blinded for the assessor.

-We also realized that in order to avoid contamination between the two groups, we would need to randomize at cluster level. Thus, the design was altered to a cluster-randomized controlled trial with assessor blinding

-We aimed at including only workers with physically strenuous work – manual skilled/semi-skilled/unskilled workers, however, already at the initial meetings with the included first municipality (Copenhagen autumn 2012), we realized that the recruitment could become too long for the timeframe of the study, why we decided to invite administrative personnel as well.

Participants:

-For practical reasons, the written material could not be handed out to each potential participant – in most municipalities the written material was made available to all workers by using the work place intranet and by placing posters and pamphlets in the lunch areas. Invitations to the recruitment meetings were ‘handed out’ in the same way. Interested workers did not have to make contact with their supervisor – but simply show up at the recruitment meeting.

-At all recruitment meetings, the potential participants were given the option of filling out the baseline questionnaire and the consent form right away or taking it home in the envelope. Most participants chose the first.

Inclusion:

-We aimed at including a total of 500 participants (see changes in power calculation section below) from the Capital Region and Region of Zealand.

Non-inclusion/exclusion:

-We decided to drop the exclusion criteria on pre-arrangements to leave the work place/go on leave because we thereby would lose data e.g. from months of participation prior to leaving.

-It turned out that we were not able to obtain information on the participants on sick leave due to other causes than back pain – due to regulations on public work places that the work place are not allowed to share personal information on employees. Therefore, we were not able to track participants on sick leave systematically. If the worker were using their work phone, we would not be able to contact them during sick leave. The workers using private phones often made us aware through the monthly assessments.

Page 5

The intervention:

The intervention content took a more detailed form during the autumn 2012 where we had close correspondence with the Norwegian research team behind the prior studies. Thus, the content and the approach in general are more thoroughly described in later versions of the protocol and in the draft for the paper.

Page 6

-In terms of the effect parameters, we made one major alteration during the first half of the study: we decided to have Functional level and Sickness Absence as the primary outcomes and deem work ability to become a secondary outcome. It had been an ongoing discussion for quite some time, which outcomes were the most important ones for our study. Initially we thought that functional level and work ability were better outcomes that would be more realistic to influence with the highly simple intervention. However, because sickness absence had been the primary outcome in the prior Norwegian studies (with slightly more complex interventions, though), and because our work ability item was not back pain specific, we decided to have back pain–related Functional level and sickness absence as our primary outcomes. This was then also changed in the clinicaltrials.gov-record on the study.

-Although it might appear that way, the LBP-BS and the Core Outcome Measures Index (CORE), referred to in the manuscript for the article, is the same (cf. the Deyo-reference in both). The scale seemingly took another name as it was further developed and later validated.

-We were not allowed to obtain register data from the work places on the participants sick leave, why we had to rely solely on the self-reports from the participants.

-We added an extra secondary outcome just before the first baseline-assessment in November 2012: depression – measured using a single item from the Subjective Health Complains questionnaire. This is described in the manuscript. Because we then had a total of 9 questions for the follow-up assessments, we decided to collect data on the pain medication-parameter and depression-parameter every 2. Month in order to keep the monthly assessments on a total of 8 questions.

Page 7

-The time frame of the study (being a part of a PhD) did not allow us to pursue the initial goal of validating the items taken from validates scales (COMI/BBQ).

-We were not able (as planned) to hire an assistant to conduct the follow-up telephone data collections, why these were done by the primary investigator instead.

Statistics:

-We had to re-think our entire plan for the analyses as we realized that we did not have comparable groups based on the baseline-data. The study had suffered a ‘skewed’ inclusion of manual and administrative workers in the two groups, why we needed to conduct analyses that allowed us to adjust for baseline differences. Furthermore, the analyses chosen in the planning phase were not applicable with the fact that our data consisted of 12 assessments with varying people represented in the data (depending on their back pain status at each of the 12 assessments). For some reason, the statistician we consulted in the planning phase had not been aware of this. Therefore, we contacted Lars Andersen, who is very competent at statistics and master statistic software that allow for more complex analyses (SAS) and we re-did the plan for the analyses.

Page 8

Statistics:

-In the initial power calculation, we had used a SD on 1.2 – however – the literature said 1.3 – why the number of participants needed to include changed from 92 to 108. Therefore we altered the goal of 400 participants in total to 500. This would also leave more ‘space’ for drop-outs.

Blinding:

-As before mentioned, we were not able to conduct the study as observer-blinded. We did, however, blind the assessor Lars Andersen and the codes for group allocation were not ‘broken’ until the analyses were terminated.

-The process was of collecting and entering data was somewhat altered as the primary investigator had to perform more of the tasks than initially planned:

Baseline data and consent form was collected by the primary investigator who also held the recruitment meetings. She also did the initial entering of the data into a database. Afterwards, the research secretary re-did the entering of the baseline-data to check errors. The research secretary then blinded the group allocation by re-naming the groups to ‘x’ and ‘y’. Thus, neither the primary investigator nor the assessor Lars Andersen knew the group allocation during the conduction of the analyses.

-The follow-up data was initially collected by the supplier of the SMS-data collection (SMS-track.com). Because the primary investigator had to do the follow-up telephone data collections each month she had to have access to the data to see who to call. After completion of the follow-up in all 5 municipalities, the supplier provided us with an Excel-file with all the follow-up data. The data ‘cleaning’ was done by the primary investigator.

Page 9

Economical considerations:

-During the second year of the study, we managed to get a grant from The Danish Working Environment Research Fund paying the salary of the primary investigator for 1 year.
